# Automatic Carbon Dioxide-Methane Gas Sensor Based on the Solubility of Gases in Water

**DOI:** 10.3390/s120810742

**Published:** 2012-08-06

**Authors:** Raúl O. Cadena-Pereda, Eric M. Rivera-Muñoz, Gilberto Herrera-Ruiz, Domingo J. Gomez-Melendez, Ely K. Anaya-Rivera

**Affiliations:** 1 Laboratorio de Biotrónica, Departamento de Investigación y Posgrado, Facultad de Ingeniería, Universidad Autónoma de Querétaro, Centro Universitario Cerro de las Campanas S/N, Querétaro 76010, Mexico; 2 Universidad Politécnica de Querétaro, Carretera Estatal 420 S/N, El Rosario, C.P. 76240, El Marqués, Querétaro 76010, Mexico; E-Mails: domingo.gomez@upq.mx (D.J.G.-M.); karina.anaya@upq.mx (E.K.A.-R.); 3 Centro de Física Aplicada y Tecnología Avanzada Universidad Nacional Autónoma de México, A. P. 1-1010, Querétaro 76000, Mexico; E-Mail: emrivera@fata.unam.mx; 4 Facultad de Ingeniería, Universidad Autónoma de Querétaro, Centro Universitario Cerro de las Campanas S/N, Querétaro 76010, Mexico; E-Mail: gherrera@uaq.mx

**Keywords:** reconfigurable logic, gas solubility, binary gas sensor, mathematical model

## Abstract

Biogas methane content is a relevant variable in anaerobic digestion processing where knowledge of process kinetics or an early indicator of digester failure is needed. The contribution of this work is the development of a novel, simple and low cost automatic carbon dioxide-methane gas sensor based on the solubility of gases in water as the precursor of a sensor for biogas quality monitoring. The device described in this work was used for determining the composition of binary mixtures, such as carbon dioxide-methane, in the range of 0–100%. The design and implementation of a digital signal processor and control system into a low-cost Field Programmable Gate Array (FPGA) platform has permitted the successful application of data acquisition, data distribution and digital data processing, making the construction of a standalone carbon dioxide-methane gas sensor possible.

## 1. Introduction

Low-cost chemical analysis sensors can have a great impact in fields such as environmental preservation and energy efficiency. There is a growing market for this kind of sensor, especially for low-cost and reliable sensors focused on carbon dioxide (CO_2_)-methane (CH_4_) mixtures in biogas quality monitoring applications [1]. Currently, the most studied method to transform waste into energy is anaerobic digestion, which can convert a variety of wastes, such as agricultural waste from animals and plants and municipal waste, into a full energy product such as biogas [2]. The preferred technology for the analysis of single components in raw biogas, specifically to determine the CH_4_ and CO_2_ content, is optical sensors, which detect infrared absorption in the characteristic wavelengths for these molecules [3]. In the literature, biogas composition has been measured with gas infrared analyzers, such as those used by Sedlačík and Dvořáčková [2], who utilized a GASCARD II infrared gas sensor from Edinburgh Instruments [4]; Nordberg *et al.* [5], who used a Model 6500 visible/near-infrared scanning monochromator from FOSS NIRSystems [6] and Steyer *et al.* [7], who used a Siemens Ultramat 22P, which works on the principle of the nondispersive absorption of infrared light [8]. Additionally, semiconductor diode lasers for use in the mid-infrared spectral region based upon lead-salt operating near 7.8 μm have also been used for methane measurements [9]. However, there are two main drawbacks to the sensors outlined above: high cost and difficulty of installation at all biogas production sites [1]. For comparison, the cost of a commercial FTIR spectrophotometer is near $20,000 USD [10], while the estimated cost of the gas sensor described here is approximately $5,000 USD. The described sensor is also easy to build and operate.

Methods for the acquisition of biogas methane content based in a variety of measurement principles have been reported in the literature. For example, Mandal *et al.* [11] determined biogas quality using flame temperature as the measurement principle. In this case, the steady-state flame temperature was measured using a system consisting of a thermocouple probe and an analog temperature indicator. In addition, Rego and Mendes [1] and Rego *et al.* [12] described a permselective gas sensor for determining the composition of carbon dioxide-methane mixtures in the 0–100% range. The sensor consisted of a permselective membrane, a pressure transducer for measuring the permeate pressure and a needle valve for controlling the permeate outlet to the atmosphere. Furthermore, Rozzi *et al.* [13] used a thermostatically controlled cell containing 0.1 mol · L^−1^ sodium bicarbonate in which the pH was monitored by an Orion combination glass electrode and an Orion Model 601A specific ion meter. When the pH reading had stabilized, gas samples were taken using a syringe and analyzed for CO_2_ and CH_4_ content using gas-solid chromatography on molecular sieves with nitrogen as the carrier gas and a katharometer for the detector.

Carlson and Martisson [14] presented a technique to quantify variations in ultrasound pulse shape caused by interactions between the constituents of a two-component gas mixture as an alternative method to extract information concerning the molar fraction of a gas in a binary mixture. Additionally, Tardy *et al.* [15] developed a dynamic thermal conductivity sensor for gas detection based on the transient thermal response of a SiC micro-plate slightly heated by a screen-printed Pt resistance. This device was intended for specific application in the determination of the specific gases in a mixture.

Gonzalez *et al.* [16] used a device that passed the produced biogas through an Erlenmeyer flask filled with a 20% NaOH solution followed by a tube filled with soda lime pellets. The gas then passed through a Mariotte flask system containing water for the quantification of methane production. The displaced water was collected in a plastic container on a pressure sensor (QB 745, DS-Europe) for continuous monitoring of CH_4_ production.

A Field Programmable Gate Array (FPGA) is an array of basic logic blocks where the user can define its interconnectivity, making it programmable in a fully open architecture. Therefore, an FPGA provides the advantages of a general-purpose processor and a specialized circuit that can be reconfigured as many times as necessary until the required functionality is achieved. The speed and size of the FPGA are comparable with the Application Specific Integrated Circuit (ASIC), but the FPGA is more versatile and its design cycle is shorter because of its reconfigurability. FPGA applications go beyond the simple implementation of digital logic; they can be used for the implementation of specific architectures for speeding up some algorithms. A specific structure for an algorithm implemented into an FPGA could have 10–100 times higher performance than its implementation on a Digital Signal Processor (DSP) or microprocessor.

Due to the sequential processing data flow on commercially available DSPs and microprocessors, the increase in sampling rate, mathematical processing, or versatility can impose severe restrictions on processor performance. Therefore, other alternatives for signal processing must be considered to achieve real-time data acquisition and data pre-processing. Moreover, FPGA devices have been gaining market share in system on chip (SOC) applications because they can integrate processing units defined by the user and related peripheral logic in the hardware, combining open architectures that do not depend on the manufacturer or specific platforms. However, DSPs and microprocessors have a fixed sequential construction for computation, which can easily be overloaded when the processing time between samples is significantly reduced, as in high-speed control, while FPGAs have a natural parallel architecture for high-speed computation. Along with the advantages previously cited, FPGA development is performed under Hardware Description Language (HDL), making the design portable and platform independent, which is not the case for commercially available DSPs or microprocessors.

In this paper, the development of a low-cost automatic carbon dioxide-methane gas sensor based on the principle of the solubility of gaseous species in water is reported. The novelty of this work is two-fold. First, a physical principle, never used before, is applied for binary mixture quantification, drastically reducing the cost and complexity of the equipment and facilitating on-line monitoring. Second, the hardware implemented in the FPGA has the capacity for data acquisition, data distribution, data processing, data communication and control, adding functionality and autonomy to the automatic carbon dioxide-methane gas sensor and allowing it to be deployed in the field.

## 2. Experimental Section

**Figure 1.**
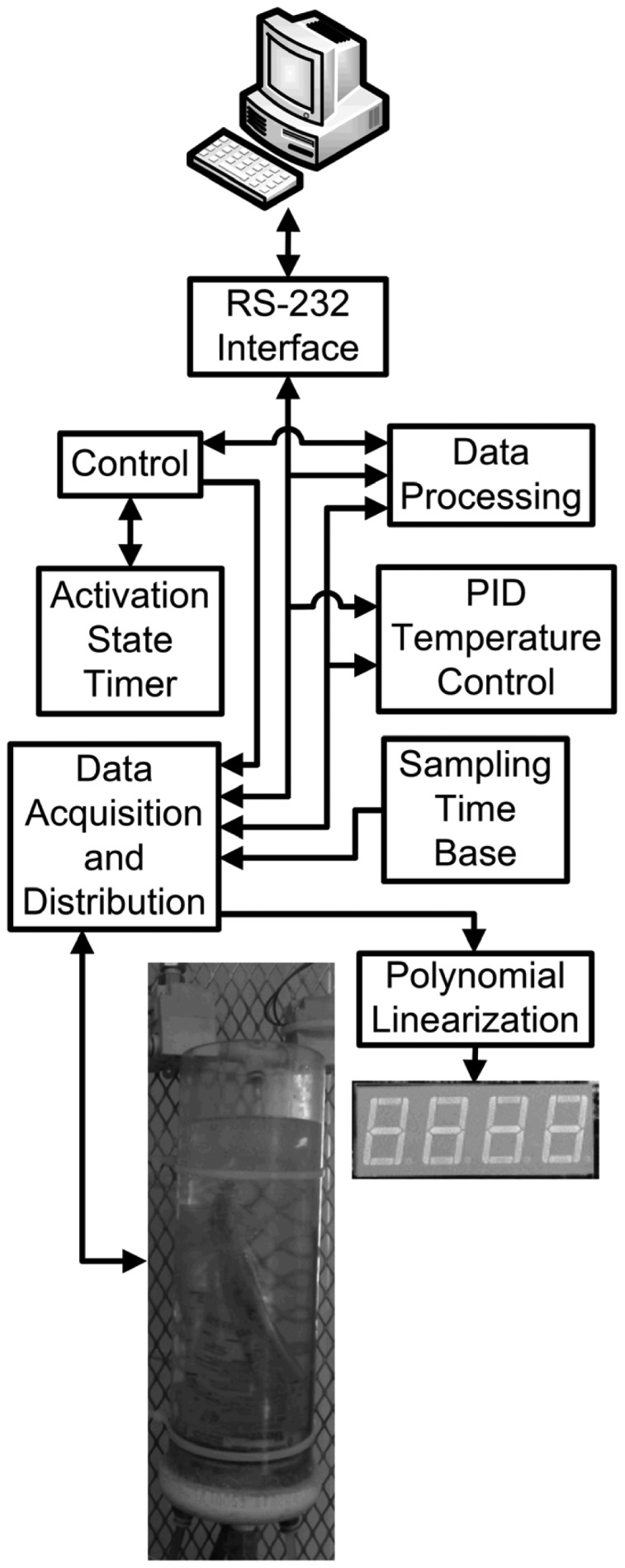
Block diagram of the carbon dioxide-methane gas sensor with the digital system.

The design of the hardware developed is divided into several components: an RS-232 Interface, an Activation State Timer, the Control, proportional integral derivative (PID) Temperature Control, Data Processing, Sampling Time Base, Data Acquisition and Distribution and Polynomial Linearization. A general block diagram of the complete digital system for the automatic carbon dioxide-methane gas sensor is shown in Figure 1.

### 2.1. Description of the Gas Sensor

**Figure 2.**
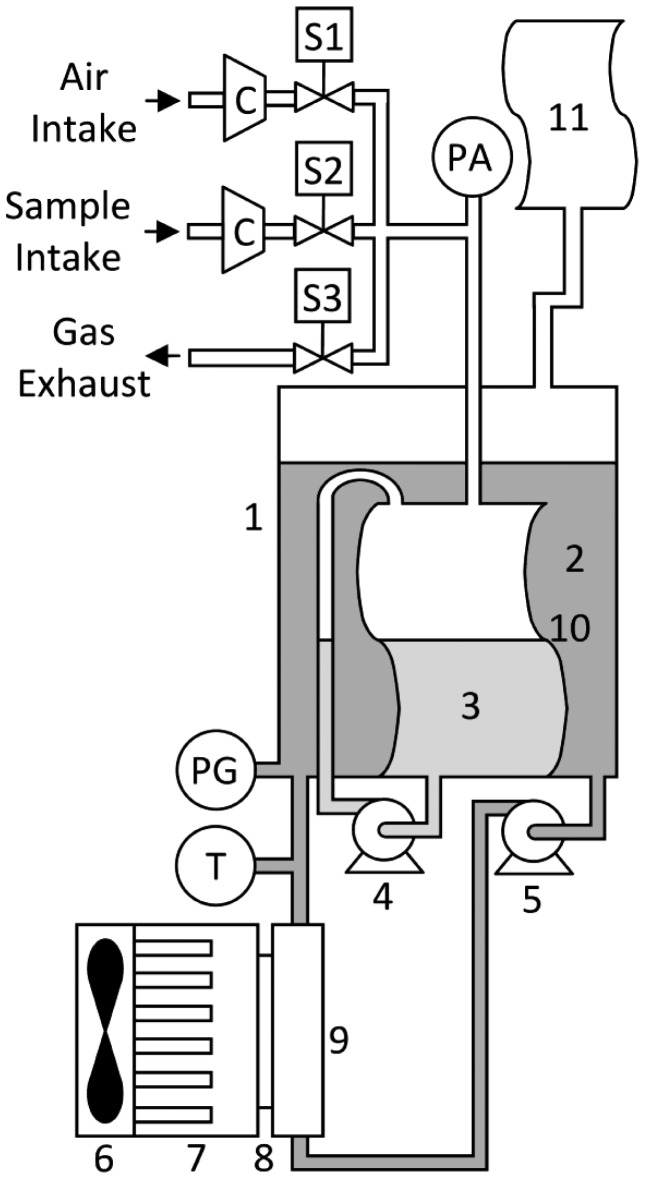
Schematic of the carbon dioxide-methane gas sensor. 1—container; 2—heat transfer fluid; 3—absorption liquid; 4 and 5—recirculation pump; 6—fan; 7—heat sink; 8—thermoelectric cell; 9—heat exchanger; 10—measurement cell; 11—flexible PVC reservoir; S1, S2 and S3—2-way solenoid valve; C—mini-compressor; PA—absolute pressure sensor; PG—gauge pressure sensor; T—temperature sensor.

To quantitatively determine the binary gas mixture, the carbon dioxide-methane gas sensor has to perform a three-stage cycle: sampling, adsorption and regeneration. In the sampling stage, the device takes in a predefined volume of gas in the measuring cell and calculates the number of moles of the binary gas mixture inside the measurement cell. In the next stage (absorption), the gas sensor removes the CO_2_ from the gaseous sample by movement-enhanced contact with a fixed quantity of absorption liquid. At the end of the absorption stage, the digital system calculates the remaining number of moles and displays the methane content percentage in the sample. In the third and last stage (regeneration), the gas sensor regenerates the CO_2_ saturated absorption liquid by movement-enhanced contact with air, releasing absorbed CO_2_ to the atmosphere. Figure 2 depicts the carbon dioxide-methane gas sensor, the constituent parts of which are a container (1), heat transfer fluid; (2), absorption liquid; (3), an absorption liquid recirculation pump; (4), a heat transfer fluid recirculation pump; (5), a fan; (6), a heat sink; (7), a thermoelectric module; (8), a heat exchanger; (9), a flexible measurement cell; (10), a flexible PVC reservoir; (11), an air intake 2-way solenoid valve (S1), a gaseous binary mixture sample intake 2-way solenoid valve (S2), a gas exhaust 2-way solenoid valve (S3), two mini compressors (C), an absolute pressure sensor (PA), a gauge pressure sensor (PG) used as level sensor, a temperature sensor (T) and electronics for control, data acquisition, data processing, data distribution, displaying and computer communication.

The container is composed of an acrylic tube (6.35 mm thick, 88.9 mm output diameter and 300 mm long) with two PVC caps attached to both ends. The aim of the container is to hold the measurement cell and the heat transfer fluid and to prevent heat transfer fluid evaporation loss to the atmosphere. The aim of the heat transfer fluid, together with the refrigeration system, is to keep the temperature of the measurement system stable. The level of the heat transfer fluid inside the container is affected by the volume inside the measurement cell, which together with a gauge pressure sensor, allows the digital system to monitor volume changes inside the measurement cell. Water was chosen as the heat transfer fluid because it has a high specific heat, is non-polluting and is abundant.

Inside the measurement cell, the absorption liquid is found. The objective of the absorption liquid is to remove the CO_2_ from the sample of the gaseous binary mixture. Water was chosen because, at 288.15 K, the CO_2_ (X_1_ = 8.21 × 10^−4^ mole fraction) is 26.29 times more soluble than CH_4_ (X_1_ = 3.122 × 10^−5^ mole fraction) [17]. Such a difference helps to efficiently separate both gaseous species, which is the physical principle proposed in this paper.

For the recirculation of the absorption liquid, a wiper washer mini-pump from ACDelco [18] was chosen because it has a small size that is suitable for this application, wide availability and low cost. This mini-pump re-circulates the absorption liquid inside the measurement cell to enhance the contact between the phases. The heat transfer fluid recirculation pump, which is also an automotive centrifugal mini-pump, re-circulates the heat transfer fluid inside the container and through the heat exchanger, promoting heat transfer from the measurement system to the atmosphere. The thermoelectric module (C1-54-2808 from Tellurex) is a semiconductor-based device that functions as a heat pump, moving heat from one of its sides to the other [19]. Among its characteristics, it can create a maximum temperature difference of 79 °C between its hot and cold sides and a maximum thermal load of 139.7 watts, achieving temperatures well below the ambient temperature. This device removes energy from the heat exchanger, pumping it to the atmosphere.

The heat exchanger is a copper plate with polished surfaces to which the thermoelectric cell can be attached, promoting heat transfer. The copper plate has holes drilled into it to allow the heat transfer fluid to re-circulate through, keeping the liquid confined but allowing heat transfer. The measurement cell is made of flexible PVC with an effective volume of 250 cm^3^, holding a fixed volume of absorption liquid necessary to absorb CO_2_ from the sample. During the sampling stage, the measurement cell also holds the volume of the gaseous binary mixture sample ready to be analyzed (100 cm^3^). The volumetric cell is also intended to serve as a barrier between the absorption liquid and the heat transfer fluid to prevent measurement error due to CO_2_ dilution into the heat transfer fluid. Although a small amount of CO_2_ permeates through the flexible PVC barrier, it is not a significant source of error. The volumetric cell film has a contact surface of 125.0 cm^2^ and a thickness of 3.3 × 10^−2^ cm. Therefore, using a CO_2_ differential partial pressure of 4.0 × 10^−1^ atm (4.13 × 10^−1^ kg middot;cm^−2^), the CO_2_ permeation through this PVC film is on the order of 1.83 × 10^−6^ cm^3^middot;s^−1^ [20]. The flexible PVC barrier actually prevents a too rapid CO_2_ dilution into the heat transfer fluid.

This carbon dioxide-methane gas sensor, with a calibrated volume of 100 cm^3^, is capable of measuring methane concentrations from 0 to 100%. Considering a worst-case scenario where the methane concentration is at a minimum, the gaseous sample is retained inside the volumetric cell for 20 min at most, so the maximum CO_2_ permeated volume reaches 2.2 × 10^−3^ cm^3^. Thus, the maximum loss is 0.0022% of the volume in every reading, an amount that can be afforded without a significant decrease in performance. The flexible PVC reservoir that permits the expansion and contraction of the measurement cell serves as a barrier to avoid direct contact of heat transfer fluid with the atmosphere, preventing evaporation and CO_2_ loss and improving measurement stability and reliability. The air intake valve (2-way solenoid) is the actuator that allows air to enter into the measuring cell each time the system is in the regeneration step. The gaseous sample intake valve (2-way solenoid) is the actuator that permits sample access to the measurement cell every time the sensor is in the sampling stage. Finally, the gas exhaust valve (2-way-solenoid) is the actuator that permits gases to exit the measurement cell every time the sensor is in the regeneration stage.

Two KPV-20A mini-compressors from Clark solutions [21] were used to move the gaseous binary mixture to be analyzed or the air for absorption liquid regeneration into the measurement cell. A US381-000005-030PA sensor from Measurement Specialties [22] was used to measure the absolute pressure inside the measurement cell. The pressure readings obtained allow the system to calculate the number of moles that enter the system. This sensor has a measurement range of 0 to 30 psi (0 to 2.109 kg · cm^−2^) of absolute pressure, with an output current range of 4 to 20 mA. A gauge pressure sensor (26PC01SMT, Honeywell) [23] was used as a level sensor of the heat transfer fluid inside the container. The gauge pressure readings properly transformed in level data represent the volume of the gas inside the measurement cell. This pressure sensor is temperature compensated, with a voltage output of 16.7 mV · psi^−1^ and a range of 0 to 1 psi (0 to 4.88 × 10^−4^ kg · cm^−2^).

The sensor used for monitoring and controlling the heat transfer fluid temperature is a LM35 temperature sensor from National Semiconductor [24]. This sensor has a measurement range from −55 to 150 °C (218.16 to 423.16 K) and a linear voltage output of 10 mV · K^−1^. The temperature of the device is maintained at 288.15 K; control is needed because CO_2_ absorption is strongly temperature dependent. It has been reported that the absorption coefficient at 288.15 K is 8.21 × 10^−4^ and at 293.15 K it is 7.07 × 10^−4^, translating to a change of 13.8% with a 5 °C temperature change [17].

In the initial state, the air intake valve and sample intake valve are closed, with both mini-compressors off, as is the absorption liquid recirculation mini-pump. The exhaust valve is open and the measurement system is ready to begin a measurement cycle. When a measurement cycle begins, the reading from the heat transfer liquid level sensor is recorded. This value is a reference from which any level change is caused by the gaseous sample and not by absorption liquid inside the measurement cell. Once the level reading is stored, the digital system closes the exhaust valve, opens the sample intake valve (S2) and the corresponding mini-compressor is turned on to pull a sample of a gaseous binary mixture with a volume of 100 cm^3^. Next, the sample intake valve (S2) closes, the mini-compressor that injects the sample is turned off, the number of moles admitted with the sample is calculated and the data obtained is stored to later calculate the CH_4_ percentage in the sample. Once the number of moles in the sample is calculated, the system turns on the absorption liquid mini-pump for 20 min. This action promotes interfacial contact between the gaseous sample and the absorption liquid, extracting CO_2_ from the sample. Once the absorption step is finished, the quantity of moles of the remaining gaseous sample inside the measurement cell is computed and the resulting data are stored.

The algebraic difference between the quantity of moles in the sample and the quantity of moles remaining after CO_2_ absorption is computed to obtain the percentage of methane content in the sampled binary mixture. The data obtained, due to the non-linear response of the system, are not ease to interpret. To overcome this situation, a polynomial linearization is also performed. The residual gas inside the measurement cell is released to the atmosphere through the activation of the gas exhaust solenoid valve (S3). To perform the regeneration cycle, the air intake valve (S1) is opened and the corresponding mini-compressor is turned on, injecting a volume of 100 cm^3^ of air into the measurement cell. At this point, the recirculation mini-pump is kept active for five minutes, after which the gas inside the measurement cell is released to the atmosphere. This cycle is repeated four times and is intended to remove CO_2_ from the absorption liquid and leave the system ready for another measurement cycle.

### 2.2. Mathematical Model

The measurement principle on which the carbon dioxide-methane gas meter is based is the difference between the water dilution coefficients of CH_4_ and CO_2_ at a given temperature (*i.e.*, a larger quantity of one gas dissolves than the other at the selected temperature). A mathematical model was thus developed to predict the theoretical behavior of this physical phenomenon and to serve as a guide into the design of the sensor. The mathematical model was also useful to validate the operational performance of the sensor. To calculate the number of moles of water needed to completely dissolve a sample consisting exclusively of CO_2_, the following equation is used:

(1) $$n_{H_{2}O}=\frac{n_{CO_{2}}-n_{CO_{2}}X_{CO_{2}}}{X_{CO_{2}}}$$

where *n_H_*_2_*_O_* is the number of moles of water, *n_CO_*_2_ is the number of moles of CO_2_ in a sample consisting exclusively of this substance and *X_CO_*_2_ is the molar fraction of CO_2_ in the water.

To determine the equilibrium that exists between a finite number of moles of a binary gas mixture (CO_2_ and CH_4_) and a finite number of moles of absorption water, the equations that describe the molar fraction of gases in water and the mole fraction of gases in the sample should be considered. The equation relating the gas dissolved in the liquid phase in contact with the gas phase should also be considered.

The following equations describe the dilution in water saturated with a binary mixture of gaseous species:

(2) $$X_{CO_{2}}P_{CO_{2}p}=\frac{n_{CO_{2}}}{n_{CO_{2}}+n_{CH_{4}}+n_{H_{2}O}}$$

(3) $$X_{CH_{4}}P_{CH_{4}p}=\frac{n_{CH_{4}}}{n_{CO_{2}}+n_{CH_{4}}+n_{H_{2}O}}$$

where *X_CO_*_2_ is the CO_2_ molar fraction in water, *P_CO_*_2_*_p_* is the partial pressure of CO_2_ (in atm), *n_CO_*_2_ is the number of moles of CO_2_ dissolved in the absorption water, *n_CH4_* is the number of moles of CH_4_ dissolved in the absorption water, *n_H_*_2_*_O_* is the number of moles of absorption water, *X_CH_*_4_ is the CH_4_ molar fraction in water and *P_CH_*_4_*_p_* is the partial pressure of CH_4_ (in atm).

Equation (2) is solved for *n_CO_*_2_ and Equation (3) is solved for *n_CH_*_4_ to obtain Equations (4) and (5), respectively:

(4) $$n_{CO_{2}}=\frac{X_{CO_{2}}P_{CO_{2}p}(n_{CH_{4}}+n_{H_{2}O})}{1-X_{CO_{2}}P_{CO_{2}p}}$$

(5) $$n_{CH_{4}}=\frac{X_{CH_{4}}P_{CH_{4}p}(n_{CO_{2}}+n_{H_{2}O})}{1-X_{CH_{4}}P_{CH_{4}p}}$$

Equations (6) and (7) describe the concentrations of CO_2_ and CH_4_ in the gaseous phase, respectively. These equations are equivalent to the partial pressure of each of the gases in the mixture:

(6) $$P_{CO_{2}p}=\frac{n_{CO_{2}g}}{n_{CO_{2}g}+n_{CH_{4}g}}$$

(7) $$P_{CH_{4}p}=\frac{n_{CH_{4}g}}{n_{CO_{2}g}+n_{CH_{4}g}}$$

where *P_CO_*_2_*_p_* is the partial pressure of CO_2_ in the mixture (in atm), *n_CO_*_2_*_g_* is the moles of CO_2_ in the gaseous phase, *n_CH_*_4_*_g_* is the moles of CH_4_ in the gaseous phase and *P_CH_*_4_*_p_* is the partial pressure of CH_4_ in the mixture (in atm).

When a gas sample is taken, there exists a finite quantity of moles of CO_2_ and CH_4_, which are in contact with a finite quantity of moles of the absorption liquid. Part of those moles in the gaseous phase will dilute into the absorption liquid until equilibrium is reached. Despite this fact, the quantity of moles of both gases remains the same. The constant quantity of moles for both of the gases is described by Equations (8) and (9), respectively:

(8) $$n_{CO_{2}m}=n_{CO_{2}}+n_{CO_{2}g}$$

(9) $$n_{CH_{4}m}=n_{CH_{4}}+n_{CH_{4}g}$$

where *_n_CO*_2_*m* is the number of moles of CO_2_ in the unaltered sample, *n_CO_*_2_ is the number of moles of CO_2_ dissolved in the absorption liquid, *n_CO_*_2_*_g_* is the number of moles of CO_2_ in the gaseous phase, *n_CH_*_4_*_m_* is the number of moles of CH_4_ in the unaltered sample, *n_CH_*_4_ is the number of moles of CH_4_ dissolved in the absorption liquid and *n_CH_*_4_*_g_* is the number of moles of CH_4_ in the gaseous phase. By solving Equation (8) for *n_CO_*_2_*_g_* and Equation (9) for *n_CH_*_4_*_g_*, Equations (10) and (11) are obtained:

(10) $$n_{CO_{2}g}=n_{CO_{2}m}-n_{CO_{2}}$$

(11) $$n_{CH_{4}g}=n_{CH_{4}m}-n_{CH_{4}}$$

To leave these equations in terms of *_n_CO*_2_, *_n_CH*_4_, *n_CO_*_2_*_m_* and *n_CO_*_4_*_m_*, Equations (10) and (11) are substituted into Equations (6) and (7), resulting in Equations (12) and (13):

(12) $$P_{CO_{2}p}=\frac{n_{CO_{2}m}-n_{CO_{2}}}{n_{CO_{2}m}-n_{CO_{2}}+n_{CH_{4}m}-n_{CH_{4}}}$$

(13) $$P_{CH_{4}p}=\frac{n_{CH_{4}m}-n_{CH_{4}}}{n_{CO_{2}m}-n_{CO_{2}}+n_{CH_{4}m}-n_{CH_{4}}}$$

Equations (12) and (13) describe the partial pressures of CO_2_ and CH_4_ in terms of *n_CO_*_2_, *n_CH_*_4_ *n_CO_*_2_*_m_* and *n_CO_*_4_*_m_*. These equations are substituted in Equations (4) and (5), resulting in Equations (14) and (15), respectively:

(14) $$n_{CO_{2}}=\frac{X_{CO_{2}}\left(\frac{n_{CO_{2}m}-n_{CO_{2}}}{n_{CO_{2}m}-n_{CO_{2}}+n_{CH_{4}m}-n_{CH_{4}}}\right)(n_{CH_{4}}+n_{H_{2}O})}{1-X_{CO_{2}}\left(\frac{n_{CO_{2}m}-n_{CO_{2}}}{n_{CO_{2}m}-n_{CO_{2}}+n_{CH_{4}m}-n_{CH_{4}}}\right)}$$

(15) $$n_{CH_{4}}=\frac{X_{CH_{4}}\left(\frac{n_{CH_{4}m}-n_{{{CH}_{4}}^{2}}}{n_{CO_{2}m}-n_{CO_{2}}+n_{CH_{4}m}-n_{CH_{4}}}\right)(n_{CO_{2}}+n_{H_{2}O})}{1-X_{CH_{4}}\left(\frac{n_{CH_{4}m}-n_{{{CH}_{4}}^{2}}}{n_{CO_{2}m}-n_{CO_{2}}+n_{CH_{4}m}-n_{CH_{4}}}\right)}$$

Equation (14) is solved for *n_CO_*_2_ and Equation (15) is solved for *n_CH_*_4_ again, resulting in Equations (16) and (17):

(16) $$n_{CO_{2}}=\frac{\left(\begin{array}{l} n_{CH_{4}}-n_{CH_{4}m}-n_{CO_{2}m}-n_{CH_{4}}X_{CO_{2}}+n_{CO_{2}m}X_{CO_{2}}-n_{H_{2}O}X_{CO_{2}}\pm \\ \sqrt{\begin{array}{l} {\left(-n_{CH_{4}}+n_{CH_{4}m}+n_{CO_{2}m}+n_{CH_{4}}X_{CO_{2}}-n_{CO_{2}m}X_{CO_{2}}+n_{H_{2}O}X_{CO_{2}}\right)}^{2}-\\ 4\left(-1+X_{CO_{2}}\right)\left(-n_{CH_{4}}n_{CO_{2}m}X_{CO_{2}}-n_{CO_{2}m}n_{H_{2}O}X_{CO_{2}}\right) \end{array}} \end{array}\right)}{2\left(-1+X_{CO_{2}}\right)}$$

(17) $$n_{CH_{4}}=\frac{\left(\begin{array}{l} -n_{CH_{4}m}+n_{CO_{2}}-n_{CO_{2}m}-n_{CO_{2}m}X_{CH_{4}}-n_{CO_{2}}X_{CH_{4}}-n_{H_{2}O}X_{CH_{4}}\pm \\ \sqrt{\begin{array}{l} {\left(n_{CH_{4}m}-n_{CO_{2}}+n_{CO_{2}m}-n_{CH_{4}m}X_{CH_{4}}+n_{CO_{2}}X_{CH_{4}}+n_{H_{2}O}X_{CH_{4}}\right)}^{2}-\\ 4\left(-1+X_{CH_{4}}\right)\left(-n_{CH_{4}m}n_{CO_{2}}X_{CH_{4}}-n_{CH_{4}m}n_{H_{2}O}X_{CH_{4}}\right) \end{array}} \end{array}\right)}{2\left(-1+X_{CO_{2}}\right)}$$

These equations have one square root each; therefore, there are two possible solutions that satisfy each of them. To make them independent of each other, Equation (16) was substituted into Equation (17) and *vice versa*. The resulting equations were then solved for *_n_CO*_2_ and *_n_CH*_4_, providing Equations (18) and (19):

(18) $$n_{CO_{2}}=\frac{\left(\begin{array}{l} \left(\begin{array}{l} n_{CH_{4}m}X_{CO_{2}}+n_{CO_{2}m}X_{CO_{2}}-n_{CH_{4}m}X_{CH_{4}}X_{CO_{2}}\\ -n_{H_{2}O}X_{CH_{4}}X_{{CO}_{2}}-n_{CO_{2}m}{X_{{CO}_{2}}}^{2}+n_{H_{2}O}{X_{{CO}_{2}}}^{2} \end{array}\right)-\\ \sqrt{\begin{array}{l} {\left(\begin{array}{l} -n_{CH_{4}m}X_{{CO}_{2}}-n_{CO_{2}m}X_{CO_{2}}+n_{CH_{4}m}X_{CH_{4}}X_{CO_{2}}\\ +n_{H_{2}O}X_{CH_{4}}X_{CO_{2}}+n_{CO_{2}m}{X_{{CO}_{2}}}^{2}-n_{H_{2}O}{X_{{CO}_{2}}}^{2} \end{array}\right)}^{2}-\\ 4n_{CO_{2}m}n_{H_{2}O}{X_{{CO}_{2}}}^{2}\left(-X_{CH_{4}}+X_{CO_{2}}+X_{CH_{4}}X_{CO_{2}}-{X_{{CO}_{2}}}^{2}\right) \end{array}} \end{array}\right)}{2\left(-X_{CH_{4}}+X_{CO_{2}}+X_{CH_{4}}X_{CO_{2}}-{X_{{CO}_{2}}}^{2}\right)}$$

(19) $$n_{CH_{4}}=\frac{\left(\begin{array}{l} \left(\begin{array}{l} n_{CH_{4}m}X_{CH_{4}}+n_{CO_{2}m}X_{CH_{4}}-n_{CO_{2}m}X_{CH_{4}}X_{CO_{2}}\\ -n_{H_{2}O}X_{CH_{4}}X_{CO_{2}}-n_{CH_{4}m}{X_{{CH}_{4}}}^{2}+n_{H_{2}O}{X_{{CH}_{4}}}^{2} \end{array}\right)-\\ \sqrt{\begin{array}{l} {\left(\begin{array}{l} -n_{CH_{4}m}X_{CH_{4}}-n_{CO_{2}m}X_{CH_{4}}+n_{CO_{2}m}X_{CH_{4}}X_{CO_{2}}\\ +n_{H_{2}O}X_{CH_{4}}X_{CO_{2}}+n_{CH_{4}m}{X_{{CH}_{4}}}^{2}-n_{H_{2}O}{X_{{CH}_{4}}}^{2} \end{array}\right)}^{2}-\\ 4n_{CH_{4}m}n_{H_{2}O}{X_{{CH}_{4}}}^{2}\left(X_{CH_{4}}-X_{CO_{2}}+X_{CH_{4}}X_{CO_{2}}-{X_{{CH}_{4}}}^{2}\right) \end{array}} \end{array}\right)}{2\left(X_{CH_{4}}-X_{CO_{2}}+X_{CH_{4}}X_{CO_{2}}-{X_{{CH}_{4}}}^{2}\right)}$$

Equations (18) and (19) describe the number of moles of CO_2_ and CH_4_, respectively, dissolved in the absorption water at equilibrium.

Finally, to obtain the response of the device based on the mathematical model, Equation (20) is applied:

(20) $$Y=\left(\frac{\left(n_{CH_{4}m}+n_{CO_{2}m})-(n_{CH_{4}}+n_{CO_{2}}\right)}{n_{CH_{4}m}+n_{CO_{2}m}}\right)(100)$$

where *Y* is the theoretical response of the device and the data represent the percent of methane content.

### 2.3. FPGA Implementation

The digital subsystem that conducts the data acquisition, data processing, data distribution, control, PC communication and local functionality was implemented in a Spartan-3 XC3S200-FT256 FPGA [25]. This device counts with 200,000 gates, twelve 18K-bit block random access memory (RAM), twelve 18 × 18 hardware multipliers and 173 inputs and outputs defined by the user. The reference clock runs at 50 MHz. Furthermore, the board counts with a four-character, seven-segment light emitting diode (LED) display that is controlled by the FPGA to display the processed data. Finally, a 9-pin RS-232 serial port was devised to establish communication with a personal computer for data acquisition purposes. A HDL was used to describe the digital subsystem. This digital subsystem description was synthesized in the FPGA and is composed of many elements (Figure 1).

The RS-232 interface module conducts communication with a PC for data acquisition and configuration purposes. The PID temperature controller module, based on a difference equation, computes the control command for the cooling system, keeping the system temperature at 288.15 K. The Activation State Timer module keeps track of the time in which solenoid valves and mini-compressors are active, reporting to the Control module the end of this time. This timer module also provides the timing necessary in each stage of the measurement sequence. The number of moles inside the measurement cell and the percentage of methane present in the analyzed sample are computed by the Data Processing module. The Sampling Time Base module dictates the rate at which the analog to digital converters (ADCs) sample and the digital to analog converters (DACs) are updated (every 1.0 × 10^−3^ s). The Data Acquisition and Distribution modules sample, quantify and encode temperature, gauge pressure and absolute pressure electronic signals. They also translate the digital command information generated by the Control module into adequate electronic signals for solenoid valves, mini-compressors, mini-pumps, the cooling system and the display. The Polynomial Linearization module performs the computation of the mathematical operations needed to linearize the raw data obtained from the device, leaving it suitable for interpretation. The displayed data is computed and updated at the end of each measurement cycle. The Control module is a finite state machine (FSM) that commands modules to execute an action or respond to stimuli from other modules to synchronize actions in every measurement cycle.

### 2.4. Statistical Characteristics

The Accuracy is the degree of closeness of measurements of a quantity to that quantity's actual (true) value. The next equations are used. With Equation (21) data average is calculated which is a part of the accuracy. In the equation *x̄* represents the average, *n* is the data number, *i* represents the index and *x* the data [26]:

(21) $$\bar{x}=\frac{\sum_{i=1}^{n}x_{i}}{n}$$

Sample variance is calculated with Equation (22), value necessary to calculate standard deviation. In Equation (22)*s*^2^ represents the sample variance, *n* is the data number, I stands for the index, *x* is the data and *x̄* is the average value obtained with Equation (21) [26]:

(22) $$s^{2}=\frac{{\sum_{i=1}^{n}\left(x_{i}-\bar{x}\right)}^{2}}{n-1}$$

Standard Deviation is calculated with Equation (23), value necessary to calculate Accuracy. In Equation (23)*s* represents the standard deviation and *s*^2^ is the sample variance value obtained with Equation (22) [26]:

(23) $$s=\sqrt{s^{2}}$$

The accuracy is calculated with Equation (24). Where *x̄* is the average value obtained in Equation (21)*t* is the critical value *t_a,v_* for t distribution, α represents the trust range 100(1 − α)%, *n* is the data number and *s* stands for the standard deviation [26]:

(24) $$\bar{x}\pm t_{\alpha /2,n-1}\cdot \frac{s}{\sqrt{n}}$$

The precision is the degree to which repeated measurements under unchanged conditions show the same results. And the next equation is used.

The Variation Coefficient describes Precision, and it is calculated with Equation (25). To calculate the Variation Coefficient, the Standard Deviation and the Average values obtained with Equations (23) and (22) respectively are used [26]:

(25) $$v.c.=\frac{s}{\bar{x}}\left(100\%\right)$$

### 2.5. Polynomial Curve Fitting

A polynomial curve fitting is made with Matlab R2009a to linerize the output of the device and improve user readiness. The command used is p = polyfit(x,y,n) that finds the coefficients of a polynomial p(x) of degree n that fits the data, p(x(i)) to y(i), in a least squares sense. The result p is a row vector of length n + 1 containing the polynomial coefficients in descending powers as shown in Equation (26) [27]:

(26) $$p\left(x\right)=p_{1}x^{n}+p_{2}x^{n-1}+\ldots +p_{n}x+p_{n+1}$$

## 3. Results and Discussion

The composition of produced biogas is directly related to digester performance and is an early indicator of digester failure [28]. The carbon dioxide-methane gas sensor described in this work represents a first step in the creation of a low-cost device for biogas quality monitoring and can be used to optimize the operating conditions of anaerobic reactors. After an exhaustive search of specialized literature, no references were found regarding the use of the principle of solubility of gaseous species in water to determine the binary gas mixture composition or the use of FPGAs [29–34] in the implementation of digital controllers, data acquisition and data processing in sensors for determining the composition of binary gas mixtures.

The water dilution coefficients of CH_4_ and CO_2_ at 288.15 K are 3.122 × 10^−5^ and 8.21 × 10^−4^ molar fraction solubility, respectively [17]. This difference is what makes it possible to separate CH_4_ and CO_2_ through dilution in water. Equation (1) was used to obtain the minimum quantity of water necessary to completely dissolve a sample of 100 cm^3^ (0.0033389 moles) of CO_2_ (worst-case measurement scenario), obtaining a quantity of 5.148 moles of H_2_O (or 92.74 cm^3^) with an atmospheric pressure of 0.789 atm (0.815 kg · cm^−2^) and a temperature of 288.15 K.

**Figure 3.**
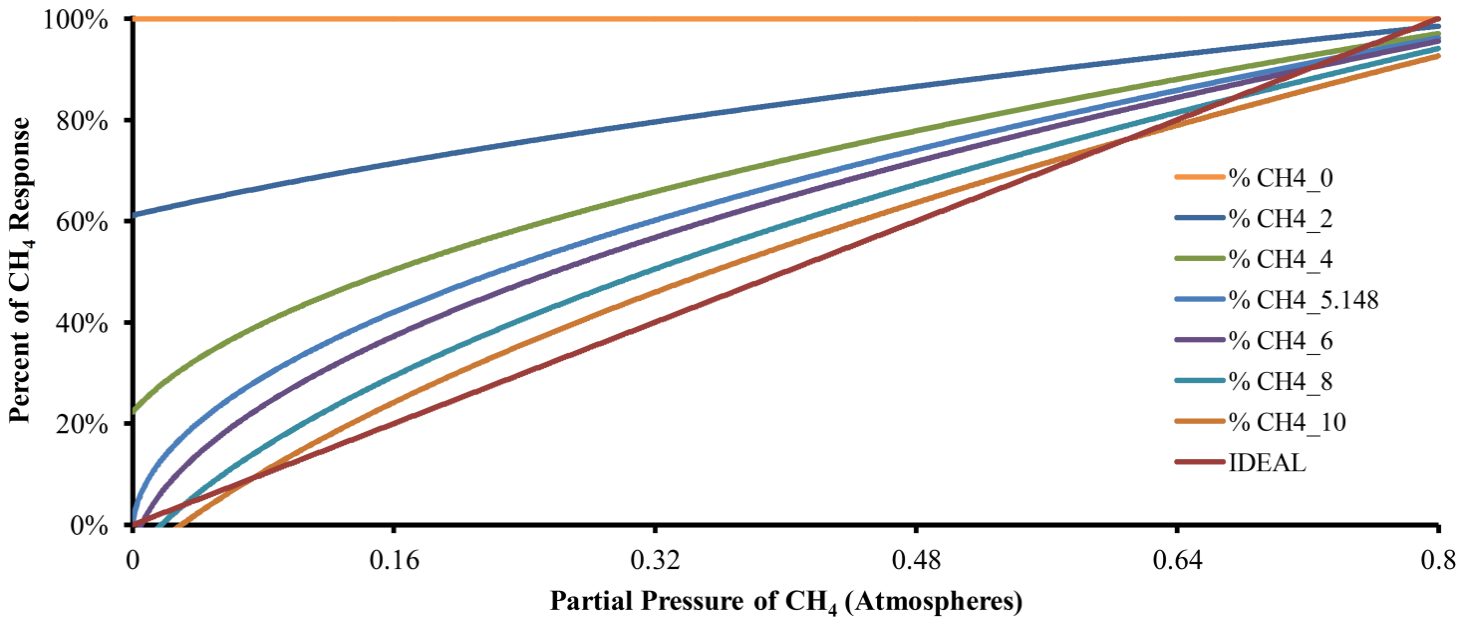
The response of the mathematical model with different quantities of absortion liquid (in moles). CH4_0 with 0 moles, CH4_2 with 2 moles, CH4_4 with 4 moles, CH4_5.148 with 5.148 moles, CH4_6 with 6 moles, CH4_8 with 8 moles and CH4_10 with 10 moles. In addition, an IDEAL plot is included for a theoretical perfect response.

Equations (18) and (19) describe the number of moles of CO_2_ and CH_4_ dissolved in the absorption liquid at equilibrium, respectively. Equation (20) uses the data generated by Equations (18) and (19) to predict the behavior of the described device. Six responses of the mathematical model with different quantities of absorption liquid in addition to an ideal or perfect response are shown in Figure 3, where CH4_0, CH4_2, CH4_4, CH4_6, CH4_8 and CH4_10 correspond to the responses with zero, two, four, six, eight and ten moles of water, respectively. CH4_5.148 is the response with the calculated number of moles of water to be used by the device to absorb a sample of 100% CO_2_ and IDEAL represents the plot of a perfect response.

When there is no absorption liquid (plot CH4_0), the data value obtained from the model kept constant along the entire range of concentrations of CH_4_ represented a sample composed of an insoluble gas. For CH4_2, the model has a theoretical quantity of two moles of absorption liquid and the curve starts near 60%. The same behavior can be observed for CH4_4, which corresponds to a theoretical quantity of four moles of absorption liquid, but now the curve starts at 20%. In both cases, such behavior indicates that the quantity of the absorption liquid was insufficient for maximum system sensitivity. The curve of CH4_5.148 shows the response of the model with 5.148 moles of absorption liquid and starts at 0%, showing the widest dynamic range of the instrument. When there are six, eight and ten moles of absorption liquid (curves CH4_6, CH4_8 and CH4_10, respectively), the system started to show a dead band in the lower concentration of CH4 that increased with the number of moles of water. However, these curves show attenuation at higher concentrations of CH4 due to over-absorption. In the last case (CH4_10), the greatest dead-band in the lower concentrations of CH4 and the greatest attenuation in the highest concentration of CH_4_ were observed, complicating the measurement at both ends of the range and improving the linearity in the middle range. In summary, changes in the amount of absorption liquid changes the device characteristics.

To evaluate the operational performance of the binary gas sensor, a variety of CO_2_-CH_4_ binary mixture concentrations were used. The concentrations used are described in Table 1, and were made by INFRA [35]. The quantity of the absorption liquid used was 5.148 moles (92.74 cm^3^), the gas sensor temperature was set at 288.15 K and the atmospheric pressure was 0.815 kg · cm^−3^.

**Figure 4.**
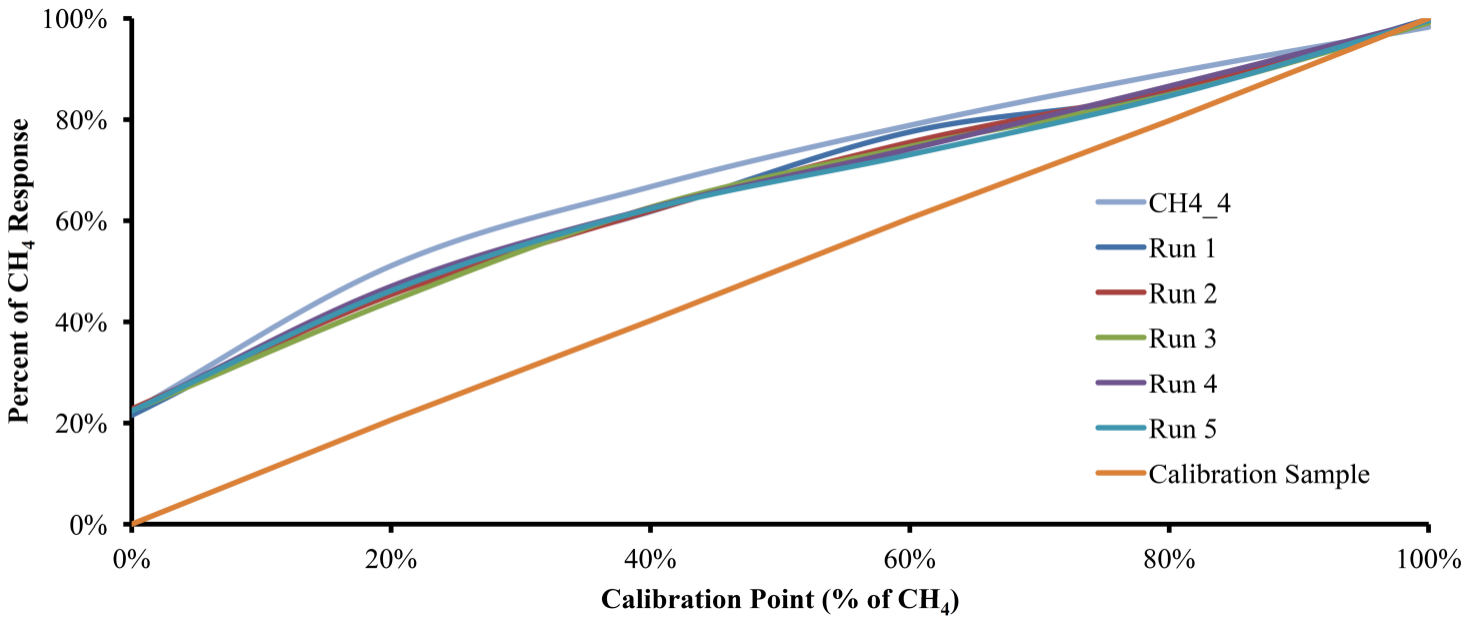
The experimental interday device response (run 1 to run 5). In addition, a plot of the mathematical model with 4 moles of absorption liquid and a plot of the Calibration Sample are shown.

Five calibration cycles were performed with each of the six gas mixtures and on different days to account for variable ambient conditions. In order to obtain the Accuracy, the critical value t was set to 2.776 and α is set to 0.05. The five runs, along with the plot of the data obtained from the model with 4.0 moles of water and a plot with the calibration gas mixture used, are shown in Figure 4. Interestingly, the experimental results with 5.148 moles of water were very similar to the results of the mathematical model with 4.0 moles of water. Such experimental behavior could be attributed to humidity content in the sample, interference of other substances diluted in the water used as the absorption liquid or to structural characteristics of the experimental device.

A polynomial linearization with Matlab R2009a allows the data obtained from the device to be adjusted for ease of interpretation. The polynomial equation used to linearize the obtained data is shown in Equation (27):

(27) $$f\left(x\right)=-12.7742x^{5}+34.5923x^{4}-35.3856x^{3}+17.9448x^{2}-3.5978x+0.2239$$

**Figure 5.**
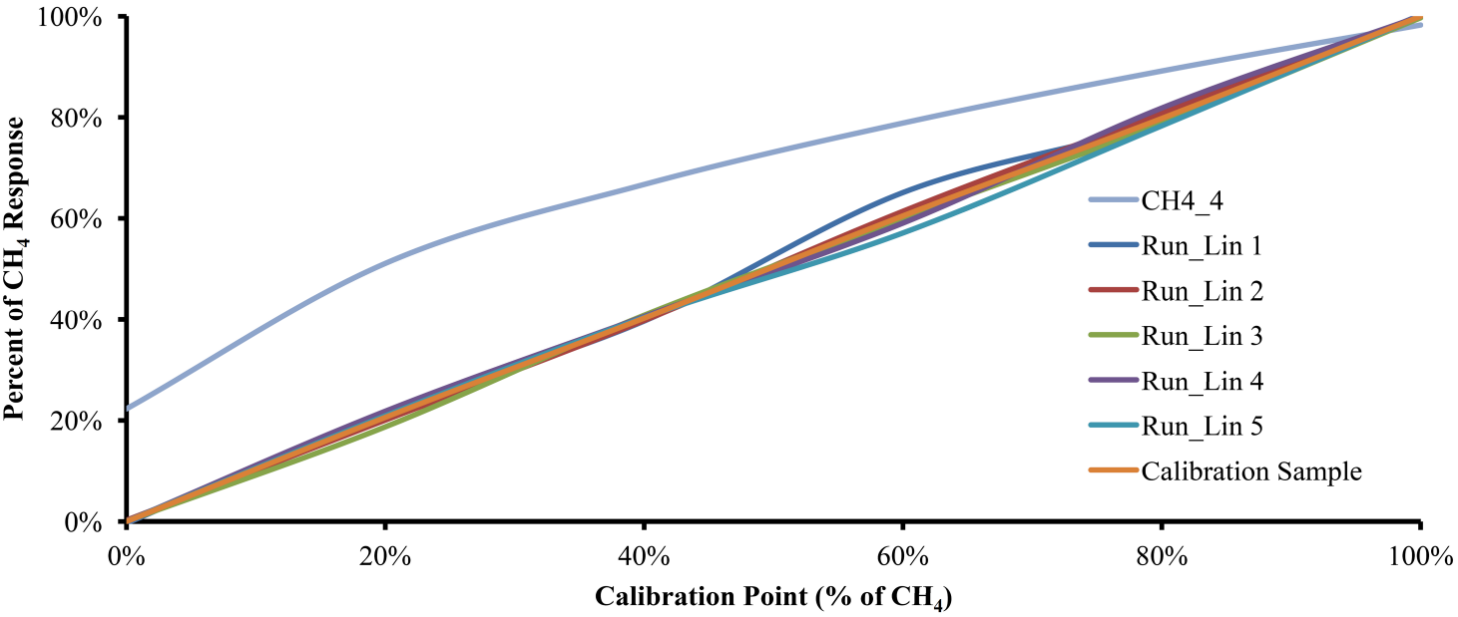
The experimental data after polynomial linearization (run 1 to run 5). In addition, a plot of the mathematical model with 4 moles of absorption liquid and a plot of the Calibration Sample are shown.

Figure 5 shows the curves of the same five runs from Figure 4 along with the plots for CH4_4 and the Calibration Sample, but using polynomial linearization Equation (21). As can be seen, the linearized plots move away from the CH4_4 curve and trend toward the Calibration Sample plot.

While there are several ways to further improve this device, it can be considered the first step toward the development of a biogas quality monitoring sensor.

## 4. Conclusions

The development of a novel, simple and low-cost automatic carbon dioxide-methane gas sensor, based on the solubility of gases in water, as the precursor of a sensor for biogas quality monitoring has been successfully completed. The device described in this work uses a novel measurement principle that makes it very simple to build and operate. The design, construction and setup of a digital processing and control system into a low-cost FPGA platform has permitted the successful implementation in a standalone carbon dioxide-methane gas sensor. The digital system developed to control the device and the data processing is very robust due to dedicated hardware implementation. The device prevents misreading due to its physical construction by minimizing CO_2_ loss, performs auto-calibration of the heat transfer fluid level at the beginning of every measurement cycle and is unaffected by the ambient temperature. The described device is a step forward in the development of a biogas quality monitoring sensor, which once completed, could be a useful tool for engineers, scientists and all those interested in following biogas quality dynamics in any kind of anaerobic digestion process.

## Figures and Tables

**Table 1.** Statistical characteristics obtained from the experimental data.

**Calibration Point**		**Device Resp. (% of CH_4_)**	**Accuracy (%)**	**Precision (%)**

**(% of CH_4_)**	**(% of CO_2_)**			
0.00%	99.99%	22.28%	±0.56%	2.04%
20.61%	79.39%	45.86%	±1.47%	2.58%
40.25%	59.75%	62.27%	±0.43%	0.56%
60.50%	39.50%	74.99%	±2.09%	2.24%
79.79%	20.21%	85.48%	±1.01%	0.95%
99.99%	0.00%	99.50%	±0.35%	0.28%
